# Optimized heterologous transfection of viable adult organotypic brain slices using an enhanced gene gun

**DOI:** 10.1186/1756-0500-6-544

**Published:** 2013-12-19

**Authors:** Jason Arsenault, John A O’Brien

**Affiliations:** 1 Neurobiology Division, MRC Laboratory of Molecular Biology, Francis Crick Avenue, Cambridge CB2 0QH, UK

**Keywords:** Organotypic brain slices, Vibroslicer, Gene gun, Biolistic transfection, Nanoparticles, Tissue slicer

## Abstract

**Background:**

Organotypic brain slices (OTBS) are an excellent experimental compromise between the facility of working with cell cultures and the biological relevance of using animal models where anatomical, morphological, and cellular function of specific brain regions can be maintained. The biological characteristics of OTBS can subsequently be examined under well-defined conditions. They do, however, have a number of limitations; most brain slices are derived from neonatal animals, as it is difficult to properly prepare and maintain adult OTBS. There are ample problems with tissue integrity as OTBS are delicate and frequently become damaged during the preparative stages. Notwithstanding these obstacles, the introduced exogenous proteins into both neuronal cells, and cells imbedded within tissues, have been consistently difficult to achieve.

**Results:**

Following the *ex vivo* extraction of adult mouse brains, mounted inside a medium-agarose matrix, we have exploited a precise slicing procedure using a custom built vibroslicer. To transfect these slices we used an improved biolistic transfection method using a custom made low-pressure barrel and novel DNA-coated nanoparticles (40 nm), which are drastically smaller than traditional microparticles. These nanoparticles also minimize tissue damage as seen by a significant reduction in lactate dehydrogenase activity as well as propidium iodide (PI) and dUTP labelling compared to larger traditional gold particles used on these OTBS. Furthermore, following EYFP exogene delivery by gene gun, the 40 nm treated OTBS displayed a significantly larger number of viable NeuN and EYFP positive cells. These OTBS expressed the exogenous proteins for many weeks.

**Conclusions:**

Our described methodology of producing OTBS, which results in better reproducibility with less tissue damage, permits the exploitation of mature fully formed adult brains for advanced neurobiological studies. The novel 40 nm particles are ideal for the viable biolistic transfection of OTBS by reducing tissue stress while maintaining long term exogene expression.

## Background

Organotypic brain slices (OTBS) are viable segments of brain tissue that can be cultured *in vitro*. Improvements in brain slice methodology are required to permit this strategy to be utilized more frequently for a wide range of biological applications. Of particular advantage is the fact that OTBS maintain many characteristics of *in vivo* biology including functional synaptic circuitry and also preserve local brain architecture [1,2]. Brain slices are increasingly being used for both basic and applied research, and have been proven successful for a number of pharmacological and genetic manipulations that investigate particular neurobiological functions [3-7]. Many biological questions can’t be sufficiently addressed using cell cultures while whole animal *in vivo* studies aren’t permissive to numerous biotechnological assays [6,7]. OTBS maintain advantages of both.

OTBS have successfully been established on a variety of brain regions including hippocampus, hypothalamus, striatum, cortex, spinal cord, and cerebellum [8-14]. For further reading on the full range of OTBS regions studied please read the review by Lossi *et al*., 2009 [2]. In addition, a range of tissue slice co-cultures have also been reported, which allow the assessment of neuronal intercommunication across brain regions. The utilities of these co-cultures have been well established with the examples of hippocampal, cortico-spinal, and cortico-striatal preparations [15-19] as well as testing OTBS under the effects of other cells such as carcinomas [20]. Several methods have been developed to maintain thin OTBS under long-term culture [21,22] based on the use of “roller tubes” or Maximov-type chambers, although preparing reproducible cultures using the conventional roller tube technique is especially demanding, and such cultures often display considerable experimental variability. Following these, a membrane interface culture method was described [23], which allows straightforward solution changes and easy access to the slices. This can be very advantageous, and many studies now use a modification of this membrane interface method. For a more indebt historical review of alternative neuronal culture methods please read Millet and Gillette, 2012 [1]. However, the preparation of experimentally reliable slices is still a major obstacle, and successfully viable adult OTBS are exceptionally rare [2,24-26].

Another major hurdle in the consistent use of OTBS is their intransigence with DNA transfection. Terminally differentiated cells such as neurons are difficult to transfect themselves, while cells within tissue aren’t easily exposed to the administered reagents. A potential solution to this is biolistic transfection, where genetic material, coated onto micro or nanoparticles, is fired through tissue, propelled by the pressurized gas of a “gene gun” [27]. This, however, can cause considerable tissue damage, exacerbating what might already have arisen from the handling of delicate OTBS. For these reasons further advancements are thus required to expand the utility of OTBS.

Here we describe an improved method of preparing adult OTBS with increased ease of preparation. These adult OTBS were then used to explore the efficiency of a modified biolistic transfection procedure using novel and less damaging, nanometer-sized DNA bound gold particles fired by a lower pressure and more precise barrel. These fired nano-particles, while concomitantly heightening the precision of the transfected area (~3 mm diameter) compared to traditional gene gun, preserve cellular viability and also elevate transfection yields within these difficult to transfect cells.

## Results

### Agarose-embedded brain slices

**Figure 1:**
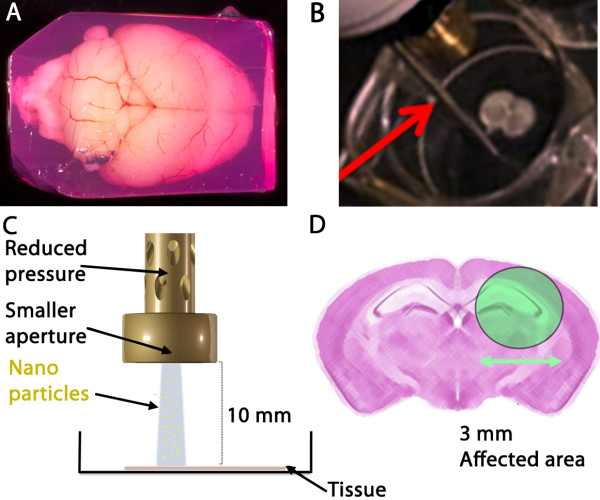
**Adult organotypic brain slice preparation and biolistic transfection. A)** The adult mouse brain were embedded in DMEM-agarose with penicillin/streptomycin. This DMEM-agarose matrix, readily suitable for handling, contains phenol red, hence the red coloration. This colour gradient also helps to see the OTBS cut by the vibroslicer. Scale bar: 10 mm. **B)** The OTBS seen in an empty well ready for transfection by biolistics. A red arrow indicates a rod support used to maintain distance and angle. **C)** Scaled model showing the height of the modified barrel over the OTBS. **D)** Distribution pattern of the scattered micro- and nanoparticles. The affected and chosen region of interest subsequently expresses the exogene [29]. This biolistic transfection method is ideally suited for adult OTBS.

To preserve the integrity of the tissue, the brains were embedded in an agarose block (Figure 1A). As the brain is placed in the ice-cold agarose solution as soon as it is removed, and the agarose block is constructed with cell-friendly Dulbecco’s modified eagle medium (DMEM) containing penicillin and streptomycin; degradation and potential contamination of the tissue were thus minimized. More importantly, the brain imbedded in the medium-agarose matrix becomes easier to slice, and these subsequent OTBS can easily be manipulated and this maintains the regional brain anatomy undamaged. Viability of the OTBS is also improved by the slicing procedure due to its minimal handling. The vibroslicer (Additional file 1: video), which we had constructed from details described by [28], is particularly appropriate for these adult brains as slicing is performed with large-amplitude and high frequency horizontal oscillations; these allow for proficient cutting of sticky myelinated fibers, of which there are an abundance in adult brain. Since vertical vibrations of the blade are minimal, typically 0.5-1.5 μm, this avoids waves of damaging compressions to the superficial tissue layers. Microscopic examination of our slices indicated that cellular and subcellular elements were largely intact 20 μm below the surface (as determined by Z scanning; data not shown). Our procedure allowed the slices to remain qualitatively viable in culture for up to 6 weeks as abundant PI negative and DAPI positive cells could still be observed within the OTBS that also maintained structural integrity (data not shown).

### Nanoparticle mediated biolistic transfection and cell survival

Efficient transfection of OTBS is difficult using traditional techniques but we have previously shown that biolistic transfection using a modified gene gun (Figure 1B-D) can result in efficient and precisely localized gene expression [27]. However, we and others have showed that biolistic transfection can cause tissue damage, which is a significant problem for these delicate adult OTBS, see [27] and refs therein.

**Figure 2:**
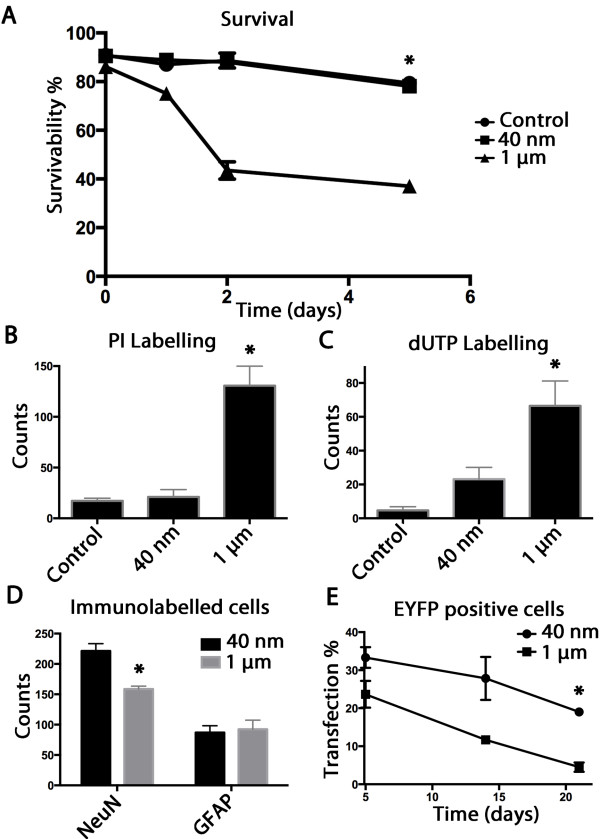
**Organotypic brain slice viability, cellular populations, and exogene expression profile. A)** Lactate dehydrogenase assay used to monitor cell viability. A significant reduction of cell viability in the 1 μm particle treated OTBS was measured using the LDH assay can be observed 5 days following treatment compared to control and 40 nm treated OTBS (n = 5, *p < 0.05). **B)** PI labelling at 5 days post transfection shows a significant elevation of the number of necrotic cells in the 1 μm treated OTBS compared to 40 nm treated and untreated OTBS (n = 6, *p < 0.05) as seen in representative brain regions. **C)** dUTP TUNEL assay at 5 days post transfection shows a slight elevation of nick end labelling in the 40 nm treated OTBS while a significant elevation in the number of dUTP positive cells compared to the untreated control can be observed in the 1 μm treated OTBS (n = 6, *p < 0.05). **D)** anti-NeuN and anti-GFAP immunolabelling at 5 days post transfection was used to quantify neuronal and glial populations respectively. A significant reduction in the number of NeuN positive cells was observed in comparable visual fields of the CA1 region of the hippocampus of corresponding coronal sections for the 1 μm compared to the 40 nm particle treated OTBS (n = 6, *p < 0.05). **E)** EYFP expression patterns seen in the 40 nm and 1 μm treated OTBS from 5 to 21 days after transfection. A significantly higher percentage of cells remained EYFP positive three weeks after transfection in the 40 nm treated OTBS compared to the 1 μm treated OTBS (n = 6, *p < 0.05) co-labelled with DAPI.

As was suggested by a previous study, the use of smaller gold particles might reduce inherent tissue stresses [29]. Here we further tested the use of nanoparticles (1/25th the scale of standard particles) to deliver the genetic material, and the OTBS displayed limited cell damage compared to 1 μm particles. Figure 2A shows total survival rates of untreated, 40 nm, and 1 μm particles treated OTBS as revealed by LDH assay. There was no difference observed between untreated and 40 nm biolistics transfected cells whereas a significant reduction in viability in the 1 μm particle treated OTBS was seen 5 days after treatment (*p < 0.05). PI labelling displayed a similar trends (Figure 2B) where the 40 nm particle treated OTBS displayed no discernable increase in the number of cells stained with this necrosis marker while the 1 μm particles significantly elevated the number of necrotic cells. dUTP labelling, a typical apoptosis marker, showed a slight increase in the 40 nm particle treated OTBS while a substantial increase in the 1 μm particle treated OTBS was observed (Figure 2C). Overall these analyses consistently indicate that the 40 nm particles are much less damaging to the OTBS than the 1 μm particles.

### Lineage quantification within the OTBS and exogene expression

**Figure 3:**
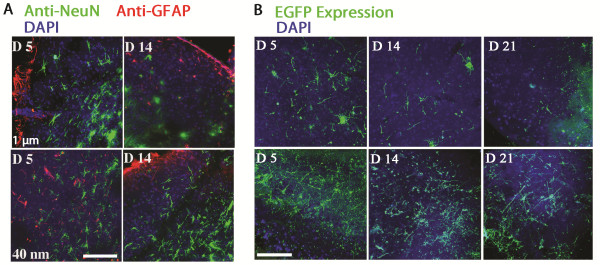
**Microscopy images of subpopulation labelling and exogene expression profiles. A)** Confocal images of the 40 nm and 1 μm treated OTBS immunolabelled with anti-NeuN, anti-GFAP, and DAPI seen at 5 and 14 days post transfection. Healthier cell morphologies can be observed in the 40 nm compared to the 1 μm treated OTBS. Scale bar: 100 μm. **B)** Confocal images of EYFP expression patterns seen in 40 nm and 1 μm transfected OTBS. A higher number of EYFP positive cells, maintaining neuronal morphology, can be observed in the 40 nm compared to 1 μm treated OTBS and this expression pattern remains longer (21 days). Scale bar: 100 μm.

In order to appropriately exploit these OTBS we measured population differences between glial and neuronal cells 5 days following transfection in the vicinity of the CA1 region. Figure 2D shows the counts of cells immunolabelled with NeuN/Fox-3, a widely used neuronal marker, in comparison to glial fibrillary acidic protein (GFAP), characteristically associated to the glial lineage. The number of NeuN positive cells counted within comparable brain regions of the corresponding coronal sections showed a significant reduction (*p < 0.05) in the number of neurons present following the 1 μm particles treatment compared to the 40 nm particles treated OTBS. However, the number of glial cells remained similar, indicating that the fragile neuronal cells were more sensitive to the 1 μm particle penetration. Figure 3A shows representative images of OTBS labelled with anti-NeuN, anti-GFAP, and DAPI at 5 and 14 days following treatment. As can be seen in Figure 3A, we have also observed GFAP positive cells proliferating along the outer edges that occurred irrespective of gold particle size used [30]. Overall, cells transfected using 40 nm particles maintain normal morphology and the expression of the immunological marker NeuN longer than the cells treated with 1 μm particles.

Transfection rates as determined by the number of EYFP positive cells over the total amount of DAPI positive cells were also more elevated in the 40 nm particle treated OTBS compared to 1 μm particles and this expression can still be abundantly observed in terminally differentiated neuronal cells (Figure 2E). This expression pattern can be seen at 5, 14, and 21 days for the 40 nm and 1 μm particle treated OTBS in Figure 3B.

### Highly detailed images of fully formed adult neurons

Biolistic transfection using 40 nm EYFP-DNA gold particles, which resulted in significantly reduced tissue damage compared to microparticles, showed brilliant expression patterns in whole cells.

**Figure 4:**
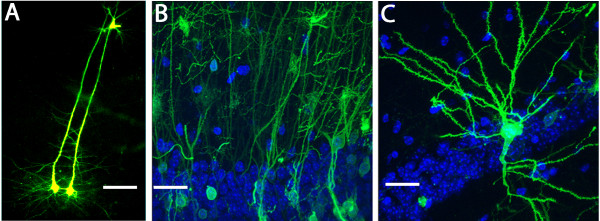
**Observing morphological characteristics in mature neurons of adult OTBS. A)** Confocal image of neighbouring pyramidal cells, which were treated with the 40 nm particles, shows long axons oriented towards the cortex as seen by EYFP expression at 5 days post transfection. Scale bar: 25 μm. **B)** Another confocal image showing a population of pyramidal cells having their soma present in the CA1 region of the brain 5 days following EYFP transfection with the 40 nm particles. The radiating arborescence of neurites formed within the adult brain can clearly be seen. Cells were also stained with DAPI. Scale bar: 10 μm. **C)** Confocal image of another pyramidal cell 5 days following transfection with 40 nm particles showing EYFP expression. The soma also rests within the densely populated CA1 region of the hippocampus while the neurites branch out towards their respective synaptic interfaces. Cells were also stained with DAPI. Scale bar: 10 μm. This transfection procedure adequately preserves the morphological characteristics and architecture of mature neurons within these *ex vivo* adult OTBS.

As can be seen in Figure 4, bright neurites of individually cells can be mapped after transfection. Figure 4A shows two parallel pyramidal cells in the hippocampus. In Figure 4B, the polarized axonal distribution shown in the CA1 hippocampal region, which is anatomically representative of this brain structure, can be clearly observed. Figure 4C shows another pyramidal cell, where fully formed mature dendrites radiate axially towards their respective synaptic interface.

## Discussion

Here we describe an improved method of preparing OTBS for biolistic transfection. The efficient medium-agarose embedded strategy, results in excellent viability due to ease of handling, and thus can be used for both immature and myelinated adult brains. We also demonstrate that using biolistic transfection with smaller, nanometer-sized particles combined with the modified gun barrel, also minimizes the damage to OTBS, which ultimately heightens exogenous protein expression inside cells that have consistently been shown refractory to DNA transfection.

Typically OTBS are prepared from the brains of animals at postnatal days 3–9. These brains show a high degree of plasticity and resistance to mechanical trauma during the preparation, which has proved important to obtain viable and healthy cultures. Although at this age basic synaptic connections have been established in a number of brain regions (e.g. in the CA1 area of the hippocampus), the mature synapses have not yet fully developed [31,32]. Thus using pup brain is less than ideal to study many aspects of synaptic physiology where adult brain would be necessary, e.g. “connectomics”, age-related neurodegeneration, adult neurogenesis [33-36].

Even in neonatal OTBS that maintain sufficient viability, there arises a gradual loss of morphological characteristics that therefore doesn’t sufficiently represent the brain regions under long-term study [37]. This loss of characteristics might be due to the more pronounced cellular de-differentiation occurring in neonatal cultures compared to older animal OTBS — a hypothesis supported by several studies [38,39]. Reproducible adult OTBS are difficult to obtain, but the DMEM-agarose embedding procedures in combination with a stable vibroslicer promote the use of adult OTBS. The vibroslicer greatly facilitates the cutting of the brain into slices which are then effortlessly separated during the slicing procedure, as opposed to a tissue chopper where difficulties in separating very thin (150 μm) and sticky adult OTBS often occurs (slices can adhere both to the blade and to themselves while using a tissue chopper). This reduced thickness of the OTBS that facilitates the absorption of nutrient from the medium was also supported by other studies [33]. Variations of culture medium supplementation for adult OTBS have also been shown encouraging [24]. The vibroslicer essentially minimizes any additional stresses to the tissue. Although our instrument was custom designed, there are now great quality commercially available vibroslicers (e.g. World Precision Instruments Model No. NVSL and Leica VT 1000S). Since it was impossible to prepare adult 150 μm OTBS using a tissue chopper, the comparison between slicing methods could not be addressed in this study. Nevertheless, ease of manipulation inherently reduces stresses to, and promotes the preservation of, these fragile tissues.

The efficient transfection of genetic material into OTBS has tremendous potential, yet, lacking in sufficient advancement, this approach has had limited success. There are many methods of inserting DNA into cultured cells, including lipofection, electroporation, viral transduction, and microinjection [40-42]. However, none of these are ideal for OTBS, largely due to the poor DNA uptake into the nucleus of terminally differentiated cells such as neurones, and this is further compounded by the lack of access to cells deep within the tissue. Biolistic transfection overcome both hindrances, but can cause significant tissue damage, which can be exasperatingly problematic with delicate adult OTBS. Our recent work [29] suggested that nanometer sized DNA coated particles are as efficient as the widely used micrometer sized particles do deliver genetic material, yet could minimize incidental damages, suggested that this approach would be ideal for OTBS and other preparations. Our current data supports this hypothesis and shows improved viability of the cells in the OTBS following genetic delivery. These adult OTBS also showed exquisite details of neural architecture that were preserved and these can subsequently be meticulously investigated. The observed morphology of these neuronal cells can thus be analysed by various methods such as measuring neurites or using a combination of fluophores, stochastically delivered, to visualise intercellular synaptic networks [7,43,44]. Overall these results show that this improved protocol to prepare adult OTBS are ideal for biolistic transfection, that the 40 nm DNA coated gold particles minimizing cell and tissue damage, and that the exogene can remains expressed for weeks.

## Conclusions

This improved method of producing finely sliced adult OTBS for biolistic transfection and exploiting novel nanometer-sized particles allows for the efficient expression of exogenous proteins while minimizing tissue damage, which is essential to properly visualize the morphological, anatomical, and cellular functions within fully formed adult brains structures. These improvements in OTBS preparation in conjunction with the modified gene gun becomes a useful platform for *in vitro* transfection of *ex vivo* organotypic slices and enhances their possible applications for subsequent genetic and biochemical manipulations. These results also encourage further translational expansion of the biolistic delivery methods for medical and bio-technological applications.

## Methods

### Brain/agarose preparation

Six week old C57 Black 6 mice were sacrificed according to ethical committee approval and their brains dissected as previously described [45]. A solution of 2% LMP agarose was prepared in Dulbecco’s modified eagles medium (DMEM) supplemented with 100 U/ml penicillin and 100 μg/ml streptomycin (P/S), and cooled (4-10°C). The freshly removed brain was then placed in a plastic mould and covered with the agarose solution; the mould was then rapidly cooled to 4°C on ice. When the agarose set (5–10 min), the medium-agarose embedded brain (Figure 1A) was removed from the mould, trimmed if necessary, and then super glued (Loctite) to a copper base platform. This was then placed into the vibroslicer chamber that contained ice-cold phosphate buffered saline (PBS: 137 mM NaCl, 2.7 mM KCl, 8.1 mM Na_2_HPO_4_, 1.76 mM, KH_2_PO_4_, pH 7.4).

### Slice preparation

Slices were prepared using a vibroslicer as previously described [28]. The vibroslicer was constructed by the engineers at the Laboratory of Molecular Biology’s mechanical workshop using these specifications [28]. Briefly, the oscillation frequency was 90 Hz, the amplitude 1.5-2.0 mm, and the cutting blade positioned at a 15° angle to the horizontal plane; the mounting block bearing the medium-agarose embedded brain was set to move at 1.7 mm/min towards the blade. Sections (150 ± 1.5 μm) were collected into a falcon containing ice-cold culture media (DMEM, 1% P/S, and 10% Fetal Calf Serum (FCS)). See Additional file 1: video. The slices were then placed into 0.4 μm, 30 mm diameter cell culture inserts (Milli Cell) in a 6 multi-well tray with culture media on the outside of the insert, and incubated in a humidified incubator at 37°C with 5% CO_2_. The coronal brain slices were carefully positioned in the centre of the insert and the insert positioned to orient the brain’s dorsal region the same way in each well.

### Preparation of micro/nano particles

Particles were prepared as described [27] using 1 μm or 40 nm diameter gold particles. Briefly, 50 μl of 0.05 M spermidine and 10 μl DNA at 1 mg/ml (pEYFP-N1; Clontech, USA) were added to 10 mg of particles. These were mixed while adding 50 μl of 1 M CaCl_2_ in 10–15 μl drops. After 5 min with occasional mixing, the supernatant was removed by centrifugation (1,000 × g for 30 s) and the gold pellet resuspended in 3.5 ml 0.075 M polyvinylpyrrollidone (PVP; Sigma). This suspension was then inserted into Tefzel tubing (0.1 mm internal diameter; Bio-Rad) using a syringe. The tubing was placed in the tubing preparation station (Bio-Rad), the gold particles were allowed to settle, and the supernatant removed with a syringe. Then the tubing was rotated to ensure an even spread of gold particles, which were subsequently dried under nitrogen flow. To create bullets, the tubing was cut using a tubing cutter (Bio-Rad) into 1 cm lengths, which were either immediately inserted into the gene gun cartridge, or kept desiccated at 4°C until required.

### Biolistic transfection

Brain slices were transfected with biolistics as previously described [29] using a gas pressure of 50 psi at a distance of 10 mm (Figure 1B and C). Briefly, an alignment rod, which can be placed alongside the wall of the well periphery, was used as a distance and angle control. A stand support was also used to help maintain perpendicular alignment. Modified gene gun barrel was constructed by, and can be obtained from the MRC-LMB mechanical workshop. The distribution pattern was aimed to concentrate around the CA1 area of the hippocampus (Figure 1D). The affected diameter was previously determined to be approximately 3 mm [29].

### Survival assays and confocal microscopy

OTBS were then cultured for a further 24 h, or as otherwise noted. Lactate dehydrogenase (LDH) assay was used to determine cell survival following treatments according to manufacturers protocol (Clontech). Briefly, the colorimetric conversion of a tetrazolium salt added to the culture medium supernatant that absorbs at 490 nm is proportional to the number of lysed cells. The signal was normalized to untreated OTBS. Triton X-100 was used as a positive assay control to define maximal absorbance (i.e. equal to 0% survival). Survival was calculated as 1 - (sampled medium - negative control)/(positive control - negative control) and presented as a%.

As the epicentre of biolistic delivery was aimed to the CA1 region, this regio-selective targeting was confirmed by EYFP signal. Targeting using the modified barrel was shown very reproducible from a 10 mm targeting distance using our method, yet any misaligned OTBS delivery was excluded from the subsequent analyses. Damaged cells were also identified using a Terminal deoxynucleotide transferase dUTP nick end labelling (TUNEL) assay (Click-iT® TUNEL Alexa Fluor® Imaging Assay; Invitrogen) [46], which detects fragments of DNA from apoptotic and necrotic cells. dUTP solution was added to the OTBS as per manufacturers protocol. Propidium Iodide (PI) was also used to monitor the number of exposed nuclei. A final concentration of PI of 1.5 μM was added to the OTBS and incubated for 30 min then washed three times with PBS.

The slices were subsequently fixed with 4% paraformaldehyde (PFA; Sigma-Aldrich). For lineage differentiation, antibodies rabbit anti-NeuN (Merck; MAB377) and guinea pig anti-GFAP (Abcam; 4674) at 1:1000 and 1:500 dilutions respectively were incubated with the OTBS for 1 h. Following three PBS washes, the secondary donkey alexa fluor 488 anti-rabbit (Life technologies) and goat alexa fluor 547 anti-guinea pig (Life technologies) were used at 1:1000 and incubated 30 min. Cells were counterstained with diamidino-2-phenylindole (DAPI) for 30 min then washed three times with PBS. Following corresponding labelling OTBS were mounted in Vectashield. Brain slices were examined using an upright Bio-Rad Radiance 2100 confocal microscope and the numbers of labelled cells were counted. Only comparative brain areas were evaluated between conditions and the same magnification, pinhole size (1 AU), and gain intensity were maintained for each test. Labelled cells were counted from confocal images taken completely within these brain regions.

### Statistical analysis

Experiments were performed in at least three independent experiments. Results are presented as mean ± standard deviation (SD). Data analysis was performed using Graphpad Prism 5.0 (La Jolla, USA). The unpaired 2-tailed Student’s *t* test was used for comparison of the average total amount of counted cells per representative image or the average absorbance measurements. A *p* value of < 0.05 was considered statistically significant.

## Competing interests

John O’Brien holds patents on the improved gene gun barrel. US patent number 10/380, 452 and European patent number 01974517.3.

## Authors’ contributions

JOB carried out the biolistic transfections and brain slice experiments, helped design the study, and contributed to writing the manuscript. JA wrote the manuscript and helped design the study. Both authors read and approved the final manuscript.

## Supplementary Material

Additional file 1 **Organotypic brain slice preparation using the vibroslicer.** The brain mounted inside the DMEM-agarose matrix was glued onto the copper platform. The blade was then properly aligned as described in the methods section. Ice-cold PBS containing P/S was added to the recipient covering both the blade and imbedded brain. Once activated, the vibroslicer, at predetermined slicing amplitude and frequency, cuts OTBS of precise thickness. These OTBS, contrasted by the phenol red in the DMEM, can easily be seen. They are then lifted by the blade, scooped, and transferred to the appropriate medium. Click here for file
